# Epigenomic Profiling of Epithelial Ovarian Cancer Stem-Cell Differentiation Reveals GPD1 Associated Immune Suppressive Microenvironment and Poor Prognosis

**DOI:** 10.3390/ijms23095120

**Published:** 2022-05-04

**Authors:** Lin-Yu Chen, Rui-Lan Huang, Po-Hsuan Su, Ling-Hui Chu, Yu-Chun Weng, Hui-Chen Wang, Hung-Cheng Lai, Kuo-Chang Wen

**Affiliations:** 1 Department of Obstetrics and Gynecology, Shuang Ho Hospital, Taipei Medical University, New Taipei City 23561, Taiwan; maple916chen@gmail.com (L.-Y.C.); gyntsgh@gmail.com (R.-L.H.); pohsuansu@gmail.com (P.-H.S.); 18418@s.tmu.edu.tw (L.-H.C.); kxther@yahoo.com.tw (Y.-C.W.); hclai30656@gmail.com (H.-C.L.); 2 Translational Epigenetics Center, Shuang Ho Hospital, Taipei Medical University, New Taipei City 23561, Taiwan; 3 Department of Obstetrics and Gynecology, School of Medicine, College of Medicine, Taipei Medical University, Taipei 11031, Taiwan; egg-0420@yahoo.com.tw

**Keywords:** epigenetics, cancer stem cells, epithelial ovarian cancer, prognostic biomarker

## Abstract

Intraperitoneal metastasis is a challenging clinical scenario in epithelial ovarian cancer (EOC). As they are distinct from hematogenous metastasizing tumors, epithelial ovarian cancer cells primarily disseminate within the peritoneal cavity to form superficially invasive carcinomas. Unfavorable pharmacokinetics for peritoneal tumors and gut toxicity collectively lead to a narrow therapeutic window and therefore limit the opportunities for a favorable clinical outcome. New insights into tumor metastasis in the peritoneal microenvironment are keenly awaited to develop new therapeutic strategies. Epithelial ovarian cancer stem cell (OCSC) seeding is considered to be a critical component of the peritoneal spread. Using a unique and stepwise process of the OCSC differentiation model may provide insight into the intraperitoneal metastasis. The transcriptome and epigenome of OCSC differentiation were characterized by expression array and MethylCap-Seq. The TCGA, AOCS, and KM-Plotter databases were used to evaluate the association between survival outcomes and the methylation/expression levels of candidate genes in the EOC datasets. The STRING database was used to investigate the protein–protein interaction (PPI) for candidates and their associated genes. The infiltration level of immune cells in EOC patients and the association between clinical outcome and OCSCs differentiation genes were estimated using the TIDE and TIME2.0 algorithms. We established an EOC differentiation model using OCSCs. After an integrated transcriptomics and methylomics analysis of OCSCs differentiation, we revealed that the genes associated with earlier OCSC differentiation were better able to reflect the patient’s outcome. The OCSC differentiation genes were involved in regulating metabolism shift and the suppressive immune microenvironment. High *GPD1* expression with high pro-tumorigenic immune cells (M2 macrophage, and cancer associated fibroblast) had worst survival. Moreover, we developed a methylation signature, constituted by *GNPDA1*, *GPD1*, *GRASP*, *HOXC11*, and *MSLN*, that may be useful for prognostic prediction in EOC. Our results revealed a novel role of epigenetic plasticity OCSC differentiation and suggested metabolic and immune intervention as a new therapeutic strategy.

## 1. Introduction

Epithelial ovarian cancer (EOC) is a heterogeneous disease and a lethal gynecological malignancy. Because of the vague symptoms of early EOC and the lack of effective screening methods, more than 70% of patients with EOC have already reached the advanced stage by the time they are diagnosed. At this late stage, a disseminated tumor within the peritoneal cavity and upper abdomen is a prevalent issue. Primary debulking surgery can significantly lengthen progression-free and overall survival in EOC. However, more than 30–40% of patients do not achieve minimal- or no-residual-tumor status as a result of debulking surgery [1]. In addition to incomplete debulking surgery, the intra-abdominal spread and establishment of distant metastases are likely to cause the failure of adjuvant platinum-based chemotherapy as well as lead to tumor recurrence [2]. Up to 85% of tumors are prone to recurrence and develop resistance to first-line chemotherapy drugs [3]. Consequently, there is an urgent need to develop therapeutic strategies that target intraperitoneal metastasis and the design of prognostic models to detect and monitor the near-term risk of intraperitoneal metastasis in human EOC [4].

In EOC, metastasis can occur through intraperitoneal routes or through the vasculature, including the circulatory and lymphatic systems. Among these, intraperitoneal metastasis plays the most important role [5]. This process behaves biologically as an essential way for EOC to undergo synchronous malignant transformation at multiple foci, thereby causing intraperitoneal field cancerization [6]. EOC can spread via the peritoneum by invading malignant ascites fluid or directly metastasizing surrounding organs because the peritoneum and omentum are covered with a layer of mesothelial cells [7]. Mesothelial cells with a submesothelial extracellular matrix can promote cancerization during the initial steps of the intraperitoneal metastasis of EOC [8]. In this process, metastatic EOC cells undergo an epithelial-to-mesenchymal transition (EMT) to spread passively and undergo a mesenchymal-to-epithelial transition (MET) at a secondary site [7]. Secondly, ascites fluid facilitates the entry of EOC cells into the lymphatic system through the main drainage channels for fluid absorption in the peritoneal cavity. Lastly, EOC cells can also enter the vasculature via the left subclavian vein by the lymphatic system that possibly contributes to the presence of EOC cells in the bloodstream [9]. Accordingly, blocking the peritoneal spread of EOC is thought to be a critical target in the development of new therapeutic strategies.

One emerging model for a better understanding of intraperitoneal metastasis is to start with a pool of self-renewing malignant progenitors known as tumor-initiating cells or cancer stem cells (CSCs). CSCs can give rise to tumor metastasis and resist chemoradiotherapy [10,11]. Ovarian cancer stem cells (OCSCs) exposed to stimuli within the tumor microenvironment have emerged as a key feature in driving intraperitoneal metastasis, suggesting that they could serve as suitable targets for therapy [12]. CSCs are at the top of the hierarchy of cell types in distinct stages of differentiation [13]. This paradigm shift from a stochastic model to a CSC model in tumorigenesis has profound implications in cancer diagnosis and therapies. However, the isolation and characterization of OCSCs are still controversial and challenging. The expression of various types of membrane ATP-binding cassette (ABC) transporters have been proven as conserved markers due to their role in drug resistance properties of CSCs [14]. The Hoechst dye exclusion assay has been useful in isolating the CSC or side population (SP) in various tissues without using cell surface markers [15,16]. In addition, OCSCs can also be isolated and characterized by surface markers such as CD133, CD44, CD117, etc. [17,18]. Nevertheless, the identification of an ideal model of OCSCs and the definition of their phenotype or function is quite challenging because of the heterogeneity of EOC. Furthermore, a deeper understanding of the pathobiology of OCSCs and their interaction with the peritoneum may shed light on the mechanisms that underlie the clinical behavior of intraperitoneal metastasis in EOC.

Previously, we discovered two types of EOC spheroids (SR1 and SR2) from single-cell-derived OCSCs [19]. Both types expressed stemness markers (CD44, CD133) and could self-renew and initiate tumors when fewer cells were used. SR1 could even show multiple differentiation potency including osteogenesis and chondrogenesis. Therefore, our spheroid SR1 fulfills the definition of CSCs. Although the other spheroid, SR2 has a limited translineage-differentiation ability, it still retains high tumorgenicity which is suggestive of tumor progenitors. Cancer progenitors (SR2) can be derived from CSCs (SR1). On the other hand, CSCs can also be enriched by suspension culture. This marker-free approach was a consequence of the success of neural stem-cell research in which an undifferentiated multipotent population of neural cells could be grown in suspension as neurospheres [20,21]. Similar approaches have been adapted to isolate nonadherent spheroids with stem-cell properties from normal breast tissues [22] and from other human cancer tissues [23,24]. Against this background, we reasoned that our stem-progenitor cell model may provide a prototype for the understanding of intraperitoneal metastasis in EOC.

In the present study, we used our cancer stem cell differentiation model to decipher epigenetic and transcriptional events in the mechanism of OCSC differentiation, which may provide insight into tumor spread and lead to novel therapeutic strategies.

## 2. Results

### 2.1. Establishment of an Epithelial Ovarian Cancer Differentiation Model

We established an epithelial ovarian cancer differentiation model using OCSCs that exhibited translineage-differentiation stem properties and were highly tumorigenic [19]. The OCSC differentiation model is illustrated in Figure S1A, including type I EOC stem spheres (SR1), type II progenitor spheres (SR2), and its four adherent differentiated progenies (ADs, AD1–AD4) from the CP70 cell line. For phase A, we compared the difference between SR1 and SR2. For phase B, we compared the difference between spheroid (SR1 and SR2) and early adherent cancer cells (AD1 and AD2). For phase C, we compared the difference between early adherent cancer cells (AD1 and AD2) and late adherent cancer cells (AD3 and AD4). Then, we harvested DNA and RNA for further investigation. The flow chart of candidate-gene selection is shown in Figure S1B.

### 2.2. Integrated Transcriptomics and Methylomics Analysis of OCSCs Differentiation

**Figure 1:**
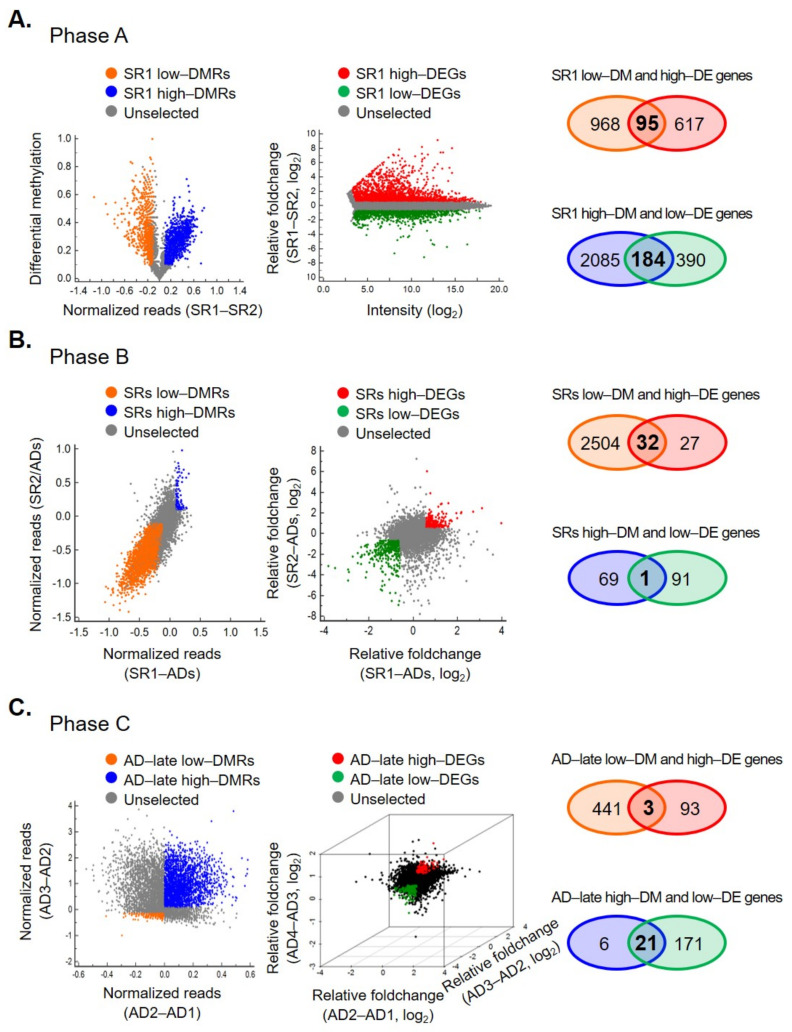
Differential DNA methylation and gene-expression profiles are divergent in three phases during epithelial ovarian cancer stem cell differentiation. The global differential methylation (left), expression fold-changes (middle), and the genes fit selective criteria (right, negative correlation between methylation and expression) in phase A, SR1 vs. SR2 (**A**), phase B, SRs vs. early adherent cells (**B**), and phase C, early adherent cells vs. late progression cells (**C**) are shown.

We hypothesized that some genes needed for, or related to, stemness might be epigenetically naïve at the stem-cell stage. When OCSCs undergo differentiation, there may be some epigenetic events that could serve as a marker for the early diagnosis of EOC as well as patient stratification of EOC prognosis. Therefore, we used a gene-expression microarray and methyl-DNA binding domain capture coupled with next-generation sequencing (MethylCap-seq) approach to identify novel epigenetically reprogrammed genes during differentiation of OCSCs. The differential methylated regions (DMRs, differential methylation changes ≥ 10%) and the differential expression genes (DEGs, │fold change│ ≥ 1.5) in three phases are shown in Figure 1 left and middle panels, respectively. Next, we combined methylome and transcriptome data and identified 95, 32, and 3 genes that were hypomethylated and associated with a higher expression in three phases, respectively. In contrast, 184, 1, and 21 genes were hypermethylated together with lower expressions (Figure 1, right panel). Following this anthology process, we identified 336 candidate genes with a methylation level that correlated negatively with the expression level and may play an important role in the differentiation of OCSCs.

### 2.3. Clinical Relevance of OCSC Differentiation Genes

**Figure 2:**
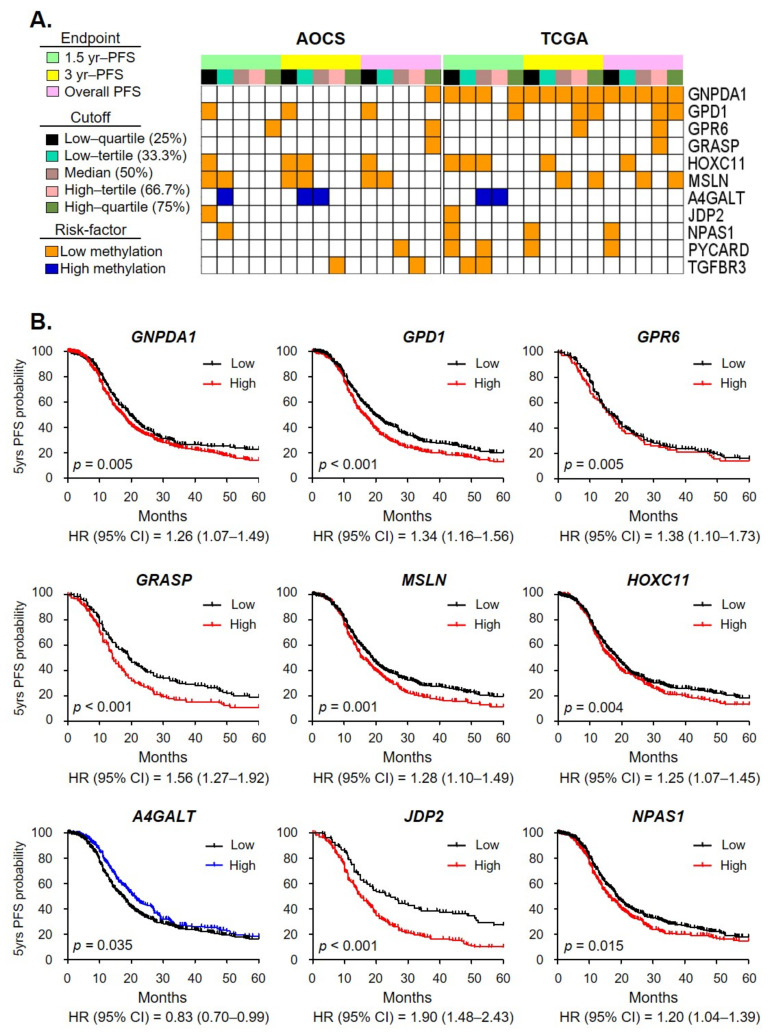
The survival relevance of OCSC differentiation genes. (**A**) Schematic of the clinical relevance of selected genes, from Figure 1, assessed by the correlation between methylation status and PFS. The prognostic significance of the DNA methylation level of each gene was tested with five cutoff points and three different endpoints, with a total of 15 criteria. Genes with low methylation associated with poor survival are highlighted with orange, while genes with high methylation associated with poor survival are highlighted with blue. Eleven gene methylation levels were significantly correlated with poor survival and were consistent in the two datasets. (**B**) The 5-year progression-free survival of nine candidate genes’ mRNA expression levels in EOC patients was examined using the KM-plotter database. The red and blue lines indicate genes from Figure 2A that were hypomethylated and hypermethylated which conferred poor survival, respectively.

To assess clinical relevance, we surveyed the prognostic significance of the DNA methylation level of each gene. To this end, we calculated the recurrent risk score (RRS) of each gene using two databases, The Cancer Genome Atlas (TCGA) and the Australian Ovarian Cancer Study (AOCS). Then, we selected the genes for which the sum of RRS was positive or negative in both databases (Figure S2). After this strict selection process involving a total of 336 candidate genes, we identified 11 genes whose methylation was significantly associated with patient survival in the two databases. Ten hypomethylated genes and one hypermethylated gene correlated with inferior survival (Figure 2A).

Next, we determined the clinical relevance of these 11 genes with gene expression using the KM-plotter database. We found nine genes (82%) that consistently showed a significant correlation of both DNA methylation and gene expression with survival in EOC patients (Figure 2B).

We then established a unique OCSC differentiation cell model, dissected by methylome and transcriptome at different phases of the patients’ disease trajectory. We found that 67% (6/9) of candidate genes were related to the patient’s prognosis in phase A, 11% (1/9) and 22% (2/9) in phase B and C, respectively. The genes that changed in the earlier phases of OCSC differentiation were better able to reflect the patient’s outcome. These findings indicate that the epigenetic plasticity of cancer stemness reflects primary tumor behavior and can predict prognosis. It has not escaped our notice that the methylation signature could be both a prognostic marker and a biomarker for individualized medicine in EOC.

### 2.4. A Methylation Signature including Metabolism Genes as a Prognostic Biomarker

**Figure 3:**
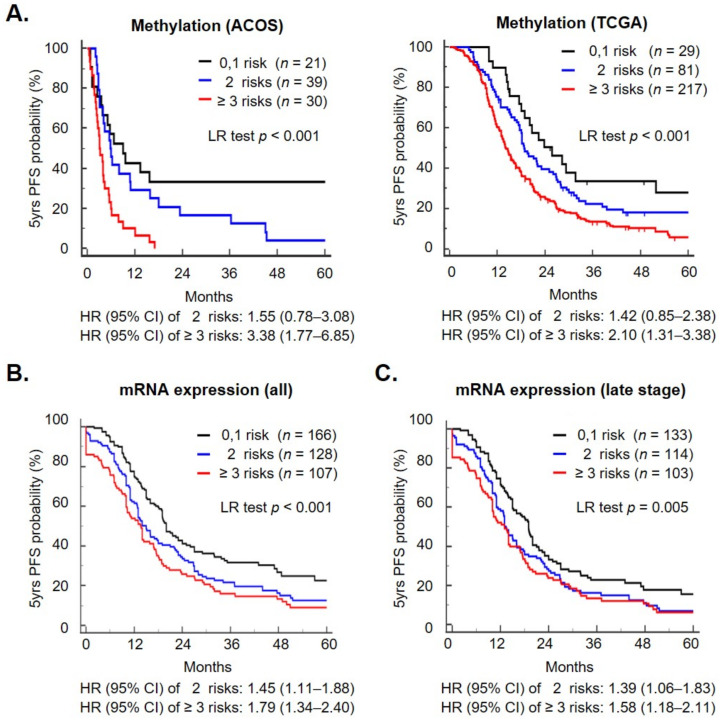
Methylation signature of OCSC differentiation genes can predict patient outcomes. The combination of the methylation status of five genes constituted OCSC methylation signature. Patients were grouped by 0–1 gene at risk (black line), 2 genes at risk (blue line), and ≥3 genes at risk (red lines) to determine the PFS. The survival was tested by methylation level of five genes with TCGA and AOCS (**A**), as well as the expression of all (**B**) and late stage (**C**) EOC patients with a KM plotter. The *p*-value was calculated using the log-rank test.

For application in clinical practice, we developed an EOC DNA methylation signature consisting of five genes (*GNPDA1*, *GPD1*, *GRASP*, *HOXC11*, and *MSLN*) that can predict patient outcomes. Patients with more than three genes at risk had a significantly shorter progression-free survival (PFS) in the two databases (Figure 3A; *p* < 0.001 by the log-rank test; ≥3 genes, 2 genes, and 0–1 gene at risk in AOCS vs. TCGA, the 60-month survival probabilities were 0% (less than 17 months), 4.2%, and 33.3% vs. 6.1%, 18.2%, and 28%, respectively). Patients classified as ≥3 genes at risk had significantly inferior PFS with a hazard ratio (HR) of 3.38 [95% confidence interval (CI), 1.77–6.85] vs. 2.10 (95% CI, 1.31–3.38) in AOCS vs. TCGA when compared with less than one gene at risk. Then, we used these five gene mRNA expression levels to analyze prognosis using the KM-plotter database [25], which integrated 15 datasets with clinical attributes, and a total of 401 EOC patients were included. In line with the findings, patients with more than three risks displayed the worst prognosis with HR = 1.79 (95% CI, 1.34–2.40), the 60-month survival probabilities were 9.0, 12.5 and 22.6% for ≥3 genes, 2 genes, and 0–1 gene at risk, respectively (Figure 3B). The risk group, TNM stage, grade, and debulk status each had a significant impact on the PFS of EOC patients as determined using a univariate analysis (Table 1). In the multivariate analysis, ≥3 genes (HR = 1.55, *p* = 0.002), late-stage (HR = 3.66; *p* < 0.001), suboptimal (HR = 1.77, *p* < 0.001) were independent predictors of PFS (Table 1, Figure S3A). For late-stage EOC patients, a Kaplan–Meier survival analysis revealed that patients with ≥3 genes at risk had a significantly reduced PFS compared with those with 0–1 gene at risk (log-rank test, *p* < 0.001, Figure 3C and Figure S3B). Taken together, we dissected the methylome and transcriptome profile of OCSCs’ differentiation and constructed a methylation signature that could predict patient prognosis outcomes.

### 2.5. A Metabolism Shift during OCSC Differentiation

**Figure 4:**
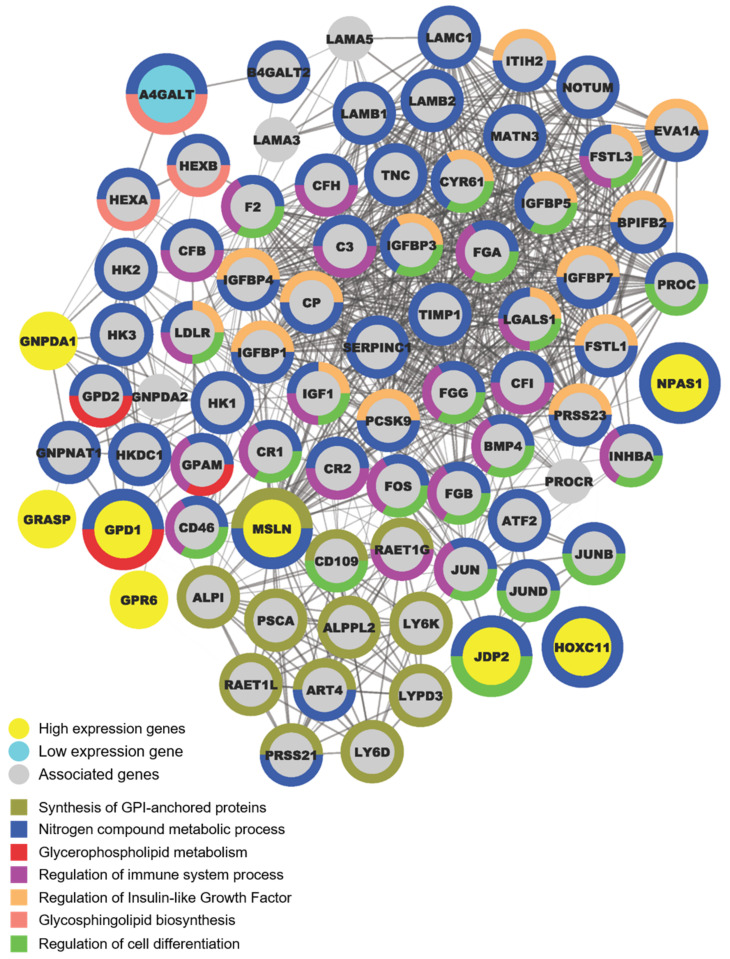
Functional annotation of OCSC differentiation genes. The network of interactions between OCSC differentiation genes and their associated genes was analyzed using STRING protein–protein interaction database. The figure highlights the connections between differentially represented genes. Proteins are represented as nodes. Yellow nodes represent candidate genes, with high expression at risk; blue node represents candidate genes, with low expression at risk; gray nodes represent other associated genes. The width of the line represents the protein–protein interaction score.

Next, we analyzed the specific pathway alterations during the differentiation of OCSCs, which may be key steps in ovarian cancer stemness, metastasis, or progression. Using protein–protein interaction (PPI) and functional annotation analysis, we found that PPI networks were mainly involved in the nitrogen compound metabolic process [nodes = 63, false discovery rate (FDR) = 6.13 × 10^−19^], including fatty acid, amino acid, and nucleotide metabolisms (Figure 4). PPI network construction revealed that the most significant enrichment occurred in the IGF signaling pathways (FDR = 7.43 × 10^−28^), which is in line with a previous report that found that the IGF-1R-AKT axis imparts functional heterogeneity in OCSCs during the acquisition of chemoresistance and the maintenance of OCSC features [26]. For phase A, most genes were associated with fatty acid, amino acid, and nucleotide metabolisms as well as cell-differentiation regulation. For phase B, genes were related to glycosphingolipid biosynthesis, while in phase C, genes were linked with posttranslational modification, the synthesis of GPI-anchored proteins, and glycerophospholipid metabolism.

### 2.6. OCSC Differentiation Genes Were Involved in Regulating Suppressive Immune Microenvironment

**Figure 5:**
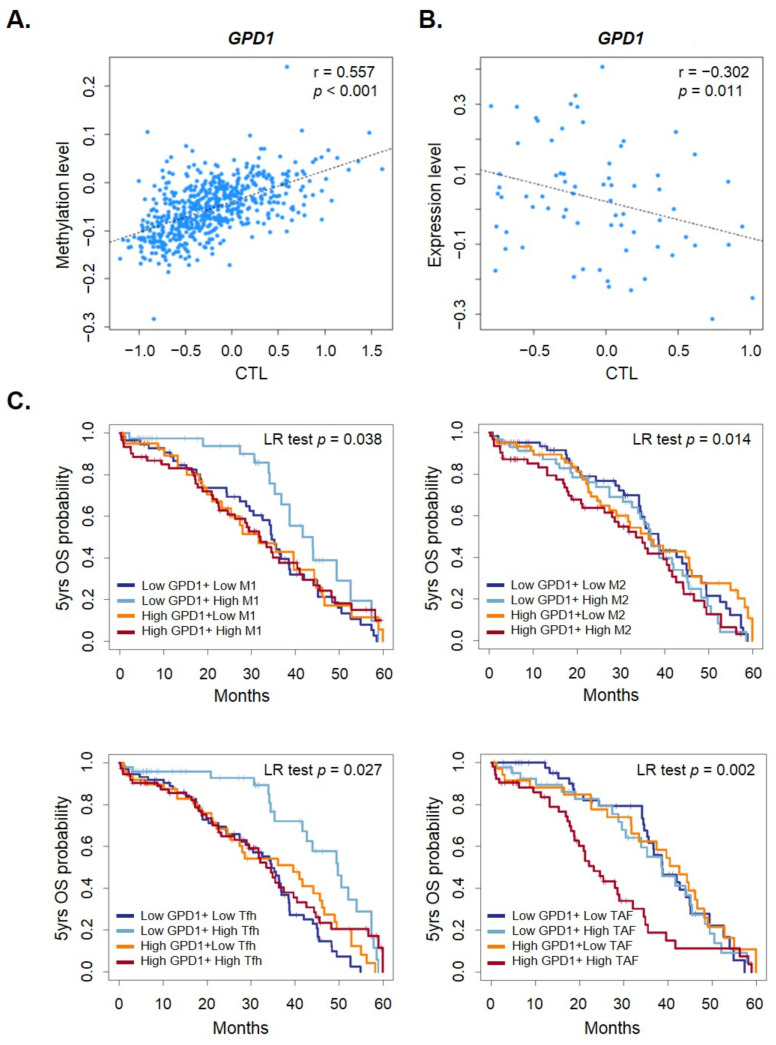
The correlation between the infiltration CTL levels and *GPD1* methylation ((**A**), *n* = 303) or GPD1 expression ((**B**), *n* = 70) level in EOC tissues was calculated using the TIDE algorithm. The plot displays the score of CTL cells for each sample against *GPD1* methylation or expression level in EOC tissue. (**C**) The 5-year overall survival of *GPD1* mRNA expression levels with M1, M2 macrophage, Tfh, and CAF in EOC patients was assessed using the TCGA database.

A new and growing recognition that metabolic changes occurring in cancer cells can impact immune cell functionality and contribute to tumor immune evasion is evident in the research [27]. The immune microenvironment is also expected to influence EOC metastasis in vivo. We therefore analyzed whether the candidate gene expression level of EOC tissue was associated with leukocytes infiltration. To assess the extent of leukocyte infiltration, we computed the correlation between these genes and cytotoxic T lymphocyte (CTL) level using the Tumor Immune Dysfunction and Exclusion (TIDE) algorithm [28]. We discovered that *GPD1*, *MSLN*, *GPR6*, and *HOXC11* methylation levels were positively associated with the CTL level (Figure 5A and Figure S4). The expression levels of three of these, *GPD1*, *MSLN*, and *GPR6*, were negative correlated with CTL level (Figure 5B and Figure S4). We found that low *GPD1* expression with high M1 macrophage (M1, anti-tumorigenic) as well as high T cell follicular helper (Tfh) had favorable survival. A high expression of *GPD1* with high M2 macrophage (M2, pro-tumorigenic) as well as high cancer associated fibroblast (CAF) led to the worst survival (Figure 5C). Consistently, the *GPD1* expression was found to be positively correlated with M2, TfH, and CAF infiltration levels using the Tumor IMmune Estimation Resource (TIMER2.0) algorithm [29] (Figure S5). A similar finding was also observed for *GPR6* (Figure S6). High GPR6 expression levels together with low M1, high M2, high CAF, low Tfh, as well as low CD4^+^ T cell and Th2 indicated the worse survival. These data suggested that high *GPD1* and *GPR6* expression levels may cause an unfavorable immune microenvironment, which leads to poor prognosis.

## 3. Discussion

In this paper, we established an OCSC differentiation model and discovered that associated molecules at an early phase of cancer stem-cell differentiation may confer the most significant role in prognosis, which sheds new light on metabolic and immune intervention for the inhibition of OCSCs.

CSCs, as a small population of cancer cells, are critical for the process of metastatic colonization as they contribute to the initial expansion of cancer cells at the secondary site [30]. For example, when CD90^+^CD24^+^ CSCs or CD90^+^CD24^+^-depleted nonCSCs are directly introduced into an MMTV-PyMT mice model through tail vein injection, only the CSCs’ population can produce lung metastases. Similarly, CD90^+^CD24^+^ CSCs isolated from pulmonary metastases are the only tumor cell population that efficiently initiates secondary metastases [31]. It is possible that because of the difference between CSCs and nonCSCs, genomic and epigenomic heterogeneities between primary and metastatic sites can also support secondary peritoneal metastasis because it is a de novo carcinogenesis occurrence. In EOC, CSCs may play a unique role in intraperitoneal metastasis given their ability to resist anoikis, which allows them to survive in nonadherent conditions within the ascites fluid in the peritoneal cavity [32]. This survival advantage in nonadherent conditions ultimately facilitates the metastasizing of stem-like cancer cells which eventually to adhere to secondary locations and generate secondary tumors. Previously, we used a microfluidic platform to study the requirement of lodgment binding under shear stress in the dynamic flow of OCSCs, supporting the recapitulation of peritoneal dissemination using the OCSC differentiation model [33]. In this study, we took advantage of different EOC spheroids (SR1 and SR2) [19], so this marker-free system can be adapted to isolate nonadherent spheroids with stem-cell properties [22,23,24] and may provide a suitable model to investigate the mechanism of intraperitoneal metastasis in human EOC.

In our OCSC differentiation model, the genes that changed in earlier stages of OCSC differentiation were more able to reflect patient outcomes suggesting that early epigenetic events of cancer stem cell differentiation confer the most significant role in prognosis. The dysregulation of specific cellular signaling pathways can result in the formation of CSCs, lead to dysregulation of stem-cell self-renewal, and result in an increased stemness of tumor cells during neoplastic transformation [34]. Most of these pathways are known to be essential for the stemness properties of normal adult stem cells, including self-renewal, differentiation, and proliferation, as well as for the development of various organs during embryogenesis. The most studied and characterized stemness pathways are NANOG, WNT/β-CATENIN, NOTCH, JAK/STAT, PI3K/AKT, and hedgehog, all of which have been shown to contribute to the formation of CSCs when dysregulated [35,36,37,38]. A positive correlation between the tumor histology/pathology grading of the majority of TCGA cases, including breast cancer (invasive ductal and lobular carcinoma), liver cancer, pancreatic cancer, cervical cancer (squamous cell carcinoma and adenocarcinoma), uterine corpus endometrial cancer, etc., with stemness indices illustrates the use of an innovative one-class logistic regression (OCLR) machine-learning algorithm to extract transcriptomic and epigenetic feature sets derived from nontransformed pluripotent stem cells and their differentiated progeny [39]. An adverse association between the stemness indices and survival was detected, which was significant for overall survival and PFS (a trend toward higher stemness index with a worse outcome) after adjusting for clinical factors. Similarly, a previous study elucidated the direct regulation by EpCAM of several reprogramming genes, including *c-MYC*, *OCT-4*, *NANOG*, *SOX2*, and *KLF4*, to help maintain the undifferentiation of human embryonic stem cells (hESCs) [40]. EpCAM may be useful as an early marker for the maintenance of the undifferentiated state of hESCs and it can be considered as an important marker in cancer and EOC [41]. In our OCSC differentiation model, we assessed the clinical relevance of nine genes with consistent results in the assessment of both DNA methylation and gene expression using the AOCS, TCGA, and KM-plotter databases. Furthermore, more candidate genes correlated with the patients’ prognosis in the early phase (phase A, 67%), compared with the later phase (11% in phase B, and 22% in phase C). A prime example of CSC plasticity during this process is their capacity to undergo EMT and MET, which links them to metastasis by allowing them to undergo fate transitions, migrate to distant locations, and then initiate proliferation once established at the metastatic site [42], whether through the ascites, the lymphatic system, or the vasculature. Importantly, the peritoneum and omentum are covered by a single layer of mesothelial cells that are generally thought to be “bystanders” of the metastatic process of EOC. CSCs can roll slower than nonmetastatic cells, thus resulting in preferential binding to the peritoneal mesothelium under ascitic fluid shear stress [33]. Sialyl Lewis^x^-P-selectin, through glycosylation cascade, mediates tumor–mesothelial adhesion in peritoneal metastasis. From our findings, three (*GPR6, GRASP, JDP2*) of the six validated genes of phase A are related to EMT and tumor migration [36,43,44]; one validated gene (*GNPDA1*) links to the glycolytic pathway and glycosylation [45]. Comprehensively, our reports revealed that those genes that changed in the earlier stages of OCSC differentiation, including those involved in EMT, were better able to act as prognostic markers.

After we analyzed the specific pathways, alterations during OCSC differentiation by PPI and the functional annotation analysis, we noticed that a metabolism shift is involved. Metabolism is the sum of chemical reactions in living cells that provides energy for life processes. The metabolisms of glucose, fatty acids, and cholesterol are often intertwined and regulated during these chemical reactions. Cancer-associated metabolism is also an important issue that may be classified into several hallmarks as follows: [1] deregulated uptake of glucose and amino acids, [2] use of glycolysis and the Krebs cycle for biosynthesis and NADPH production, [3] increased demand for nitrogen, [4] change in metabolite-driven gene regulation, and [5] metabolic interactions with the microenvironment [46]. In addition, the metabolic shift of cancer cells and tumor microenvironment involves and influences the behavior and efficiency of immune cells (both innate and adaptive immune cells), in particular, macrophages polarization may play a role in the immune escape mechanisms and is related to disease progression and poor prognosis of EOC [47,48]. In EOC, much evidence has demonstrated that adipose-rich omentum plays a pivotal role in specific metastatic tropism in the creation of the tumor microenvironment and intraperitoneal metastasis [27]. After adipocytes are reprogrammed into cancer-associated adipocytes by an EOC-derived mediator, these activated adipocytes can release various adipokines, such as IL-6, IL-8, MCP-1, and TIMP-1 that contribute to building the omental metastatic niche for EOC. Moreover, FABP4, SIK2, PI3K/AKT, etc., are also involved in the crosstalk between EOC and omental adipocytes that enhance proliferation, invasion, and metastatic progression. In addition, the adipocyte metabolism of glucose and its related pathways also plays an essential role in EOC. A case–control study using the UK-based General Practice Research Database disclosed that the adjusted odds ratio of the antidiabetic drug, Metformin, versus nonuse for EOC incidence was not significant in nondiabetic patients but was significant in diabetic patients [49]. Another study using reimbursement databases of National Health Insurance (NHI) to evaluate Metformin use in Taiwanese women with type 2 diabetes mellitus revealed that this antidiabetic drug may decrease the incidence of EOC. The overall fully adjusted HR (95% CI) for ever-users versus never-users was 0.658 (0.593–0.730). These results indicate that antidiabetic drugs such as Metformin may improve survival of EOC and may even have therapeutic effects with first-line chemotherapy through the AKT/mTOR pathway [50]. In OCSCs, some studies have shown that epithelial ovarian CSC-like spheroids bear more active glycolytic activity and involve direct glucose oxidation in the pentose cycle than parental tumor cells [51].

CD44^+^CD117^+^ OCSCs showed an elevated expression of enzymes associated with oxidative phosphorylation (OXPHOS) with higher mitochondrial ROS production, which suggests the mitochondria electron respiratory chain is specially used [52]. Here, we found that several cellular metabolic processes involved in generating macromolecular building blocks or extracting energy were activated. For example, *GPD1* is important for the regulation of the lipid metabolism. We found that the expression of many genes affecting glycerophospholipid metabolism was altered during the differentiation of OCSCs. Our unpublished data also indicate that OCSCs tend to shift from glucose oxidation to fatty acid oxidation and glutamine as their energy source. This is consistent with the finding in brain tumor stem cells that *GPD1* is essential for the maintenance of cancer stemness and the most significantly changed lipid pathway is glycerophospholipid metabolism [53]. These findings and biochemical characteristics suggest the possibility that the metabolism in OCSCs may be vulnerable to therapeutic intervention in the future.

The methylomics of EOC has been studied. However, the clinical significance of DNA methylation in EOC remains uncertain. To date, methylomics may be related to clinical prognosis or used to identify a precursor stage of EOC [54,55]. Moreover, wide-ranging genome-wide studies including epigenomics may provide more comprehensive results in analyzing prognostic biomarkers or the development of individualized medicine for EOC, compared with the investigations of single genes. A previous study collected 162 paraffin-embedded ovarian epithelial tumor tissues, including five major epithelial ovarian tumor subtypes (high- and low-grade serous, endometrioid, mucinous, and clear cell) and tumors of low malignant potential, from the Polish Ovarian Cancer study and Surveillance, Epidemiology, and End Results Residual Tissue Repository (SEER RTR) [56]. They analyzed the consensus nonnegative matrix factorization clustering of the 1000 most variable CpG sites and observed statistically significant differences of survival across these clusters. A comparison of models with and without methylation subgroups suggested that methylation subgroups added significant survival information (*p* = 0.007). In another study [57], the authors’ delineated pathways and networks altered by DNA methylation and associated with the initiation and progression of high-grade serous carcinoma [58]. With tissue from primary (platinum-naïve) tumors, recurrent platinum-resistant tumors, treated with a hypomethylating agent (HMA), and using human ovarian surface epithelial cells as normal control, genome-wide methylation profiles were determined and DNA methyltransferase expression levels were examined. Stem-cell pluripotency and cytokine networks were enriched in recurrent platinum-resistant EOC tumors, whereas drug metabolism and transport-related networks were downregulated in tumors from HMA-treated patients, compared with human ovarian surface epithelial cells. These findings provide important evidence that epigenetic reprogramming plays an important role in high-grade serous ovarian cancer (HGSOC) etiology and contributes to clinical outcomes. For our clinical practice, we identified the epithelial ovarian cancer DNA methylation signature, which can predict EOC patients’ outcomes and may relate to intraperitoneal metastasis. The present study did not only discover biomarkers for prognosis but also investigated the role of methylation in intraperitoneal metastasis.

Taken together, the results suggest that OCSC plays an important role in intraperitoneal metastasis. Understanding OCSC differentiation may further our understanding of epithelial ovarian cancer and intraperitoneal metastasis. Our results revealed a novel role of epigenetic plasticity in OCSC differentiation and suggested metabolism and immune targeting as a potential therapy for use in the future.

## 4. Materials and Methods

### 4.1. Cell Lines, CSCs and Enrichment of Spheroid Cells

The enrichment of OCSCs and establishment of single cell-forming OCSCs were performed as described previously [19]. In brief, ovarian cancer cell lines were cultured in ultra-low attachment plates (Corning®, Corning, NY, USA) in RPMI-1640 (Thermo Fisher Scientific, Waltham, MA, USA) supplied with 1% nonessential amino acids (Thermo Fisher Scientific, Waltham, MA, USA), sodium pyruvate (Thermo Fisher Scientific, Waltham, MA, USA), 10% fetal bovine serum (FBS, Biological Industries, Kibbutz Beit−Haemek, Israel), 10 μg/mL insulin (Thermo Fisher Scientific, Waltham, MA, USA), basic fibroblast growth factor (bFGF; PeproTech, Rehovot, Israel), and human recombinant epidermal growth factor (EGF; PeproTech, Rehovot, Israel). The cells were cultured in suspension, and starting from 14 days, the cultures were examined every day for sphere formation. Spheres were then dissociated and passaged at least eight times in 2 months to generate spheres, which are henceforth referred to as OCSC (SR1 and SR2) cells. For OCSCs differentiation, we transferred OCSCs from ultra-low attachment plates into standard cell culture dishes (Corning®, Corning, NY, USA) and removed growth factors bFGF and EGF. The cells were harvested and extracted for DNA and RNA at Day 8 (AD1), 21 (AD2), 25 (AD3), and 42 (AD4) after OCSCs adhesion.

### 4.2. Methylation Profiling and Gene Expression Array Analysis

The methylomics analysis of OCSC and its differentiated progeny cells was performed using MethylCap-Seq as described previously [57]. In brief, total genomic DNA was isolated and immunoprecipitated using MBD-Biotin Protein (MethylMiner™ Methylated DNA Enrichment Kit, Thermo Fisher Scientific, Waltham, MA, USA). Methylated DNA was then subjected to next-generation sequencing using a HiSeq-4000 instrument (Illumina, San Diego, CA, USA). We selected the MethylCap-seq data with uniquely mapped reads ≥40% of the total mapped reads and analyzed the methylation level for a 2K base pair region, spanning 1K base pair upstream and downstream from the transcriptional start site of the coding genes (reference genome of UCSC version hg18). Significantly different methylation patterns were analyzed using the differential methylation changes ≥10%.

An analysis of gene expression profiles of OCSC and its differentiated progeny cells was performed using SurePrint G3 Gene Expression Microarrays (Agilent Technologies, Santa Clara, CA, USA) following the manufacturer’s instructions. This array contains a total of 50,599 probes covering 27,958 RefSeq genes and 7419 lincRNAs. We filtered the absolute value of differential expression changes ≥1.5-fold. Those genes negatively correlating methylation and gene expression were selected as candidate genes.

### 4.3. Recurrent Risk Score and Survival Analysis

The TCGA (Infinium HumanMethylation27 Bead array) and AOCS (Infinium 450 K Methylation array) databases were used to evaluate the association of progress-free survival (PFS) and methylation levels of candidate genes in epithelial ovarian cancer tumor datasets.

The recurrent risk-score (RRS) was evaluated using the following five cutoff values: low-quartile (25%), low-tertile (33.3%), median (50%), high-tertile (66.7%), and high-quartile (75%)] for the methylation status of each gene to discriminate patient recurrence status at three endpoint (1.5 years, 3 years, and overall) PFS. The 15 conditions of survival analysis were examined for each gene. When a gene had low methylation and was associated with inferior survival a score of 1 was allocated; when a gene’s high methylation was associated with poor survival it was scored as −1; if methylation was not associated with survival the score was recorded as 0. The RRS summarized the score of the 15 total criteria. If both RRS were above 1 or both were less than −1 in two databases, AOSC and TCGA, were selected and survival relevance was further validated by mRNA expression level. The KM Plotter (http://kmplot.com/analysis/index.php?p=service, accessed on 22 August 2021) database was used to evaluate the association of PFS and mRNA levels of candidate genes in epithelial ovarian cancer tumor datasets [25].

### 4.4. Protein–Protein Interaction (PPI) Networks Functional Enrichment Analysis

The search tool for the retrieval of interacting genes/proteins (STRING) database [58], was complemented with heuristic methods of association and analysis, and were used to investigate the known and predicted PPI association for a candidate and its associated genes. The STRING database generates a network of PPI from high-throughput experimental data, literature and predictions based on a genomic context analysis.

### 4.5. Estimation of the Infiltration Level of Immune Cells in Tumor Tissues

The TIDE algorithm was used to predict the correlation of candidate genes’ expression and methylation level with CTL level. The TIMER 2.0 algorithm was used to estimate the immune cell types as well as the association between immune infiltrates and clinical outcome based on candidate genes’ expression.

### 4.6. Statistical Analysis of Methylation Signature

We categorized all methylation and expression values into high or low binomial codes for further statistical analysis. We also combined the binomial codes for five genes into 0–1 gene at risk, any 2 genes at risk and ≥3 genes at risk. The Kaplan–Meier analysis, log–rank test, and Cox proportional hazards model were used to estimate the survival distributions and to compare differences between the curves of PFS. A univariate Cox regression analysis calculated the hazard ratio (HR) and a 95% confidence interval (CI) for evaluating the risk of disease progression associated with the level of DNA methylation, gene expression and gene signature.

## Figures and Tables

**Table 1:** Multivariate cox regression analysis of PFS of ovarian cancer patients.

		Univariate		Multivariate	
Characteristics		Crude HR (95% CI)	*p*-Value	Adjusted HR ^1^ (95% CI)	*p*-Value
Risk group	0–1 gene	1		1	
	2 genes	1.45 (1.10–1.90)	0.008	1.46 (1.11–1.92)	0.007
	≥3 genes	1.79 (1.35–2.38)	<0.001	1.55 (1.17–2.06)	0.002
Stage	Early	1		1	
	Late	5.36 (3.06–9.36)	<0.001	3.66 (2.06–6.52)	<0.001
Grade	G1	1		1	
	G2	3.81 (1.54–9.39)	0.004	1.85 (0.74–4.67)	0.191
	G3	4.06 (1.67–9.87)	0.002	1.76 0.70–4.40)	0.227
Debulk	Optimal	1		1	
	Suboptimal	2.17 (1.72–2.74)	<0.001	1.77 (1.38–2.26)	<0.001

^1^ The HR adjusted by risk group, stage. grade, and debulk. HR, hazard ratio; CI, confidence interval.

## Data Availability

The data have been deposited in the GEO database under the accession code GSE111776.

## Supplementary Materials

The supporting information can be downloaded at the following: https://www.mdpi.com/article/10.3390/ijms23095120/s1.

## Abbreviations

AOCS	Australian Ovarian Cancer Study
CI	Confidence interval
EMT	Epithelial-to-mesenchymal transition
HR	Hazard ratio
HGSOC	High-grade serous ovarian cancer
EOC	Epithelial ovarian cancer
OCSC	Ovarian cancer stem cell
PFS	Progression-free survival
PPI	Protein–protein interaction
RRS	Recurrent risk score
STRING	Search tool for the retrieval of interacting genes/proteins
TCGA	The Cancer Genome Atlas
