# Supplementary material for: Epigenomic Profiling of Epithelial Ovarian Cancer Stem-Cell Differentiation Reveals GPD1 Associated Immune Suppressive Microenvironment and Poor Prognosis
Source: Int J Mol Sci. 2022 May 4;23(9):5120. doi: 10.3390/ijms23095120 (PMC9101898; doi:10.3390/ijms23095120)
Supplement: Supplementary file 1 [file ijms-23-05120-s001.zip › Supplementary figure_20220404_IJMS.pdf]

Fig. S1

A.

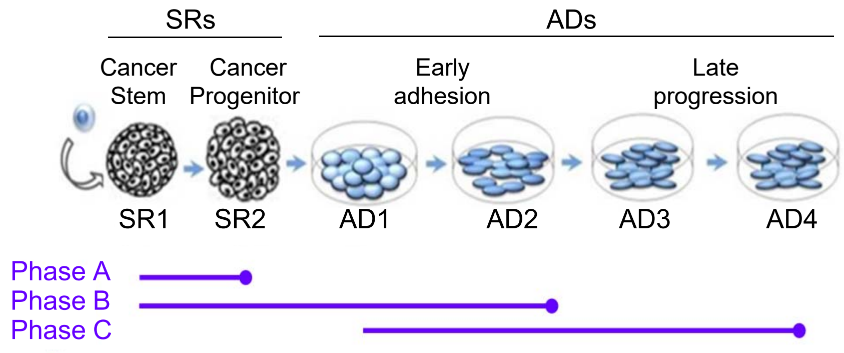

B.

Epigenomics and transcriptomics profiles in different time point cells

↓

Stage A: SR1 vs. SR2
Stage B: ADs vs. SRs
Stage C: AD-late vs. AD-early

Differentially methylated probes (DMPs) analysis

Differentially expressed genes (DEGs) analysis

Intersection of DMPs/DEGs genes

↓

Selection of candidate genes

↓

Confirmation of survival relevance using TCGA, AOCS, and KMplotter dataset

↓

Functional annotation

Evaluation of recurrent risk score (RRS) using methylation level

	Data set 1					Data set 2					
	Criteria 1	Criteria 2	Criteria 3	Criteria 4	Sum (RRS)	Criteria 1	Criteria 2	Criteria 3	Criteria 4	Sum (RRS)	
Gene 1	1	1	1	1	4	0	1	0	1	2	select
Gene 2	0	0	0	0	0	0	1	0	0	1	exclude
Gene 3	0	1	1	0	2	0	1	1	1	3	select
Gene 4	-1	-1	0	-1	-3	-1	0	0	-1	-2	select
Gene 5	0	0	0	0	0	0	-1	-1	0	-2	exclude
:											
Gene *N*	1	1	-1	0	1	1	-1	-1	0	-1	exclude

Fig. S3

A.

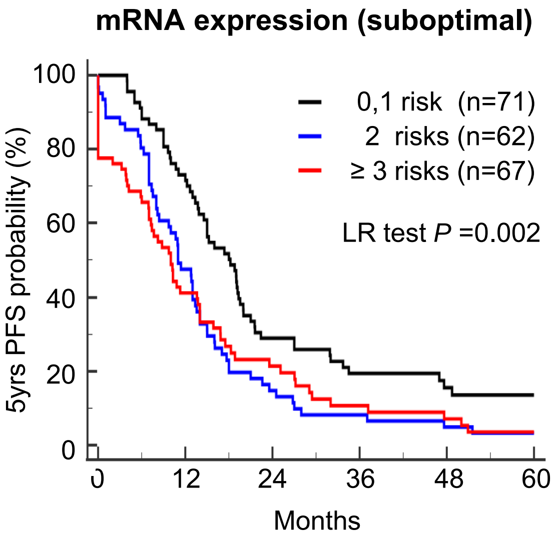

HR (95% CI) of 2 risks: 1.77 (1.23-2.53)

HR (95% CI) of ≥ 3 risks: 1.76 (1.24-2.52)

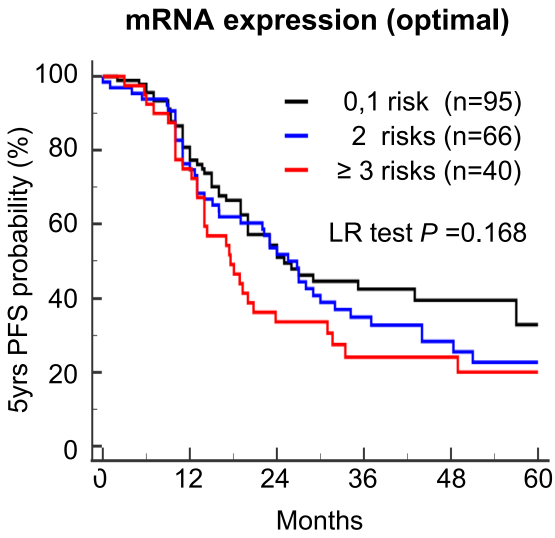

HR (95% CI) of 2 risks: 1.22 (0.82-1.81)

HR (95% CI) of ≥ 3 risks: 1.54 (0.95-2.51)

B.

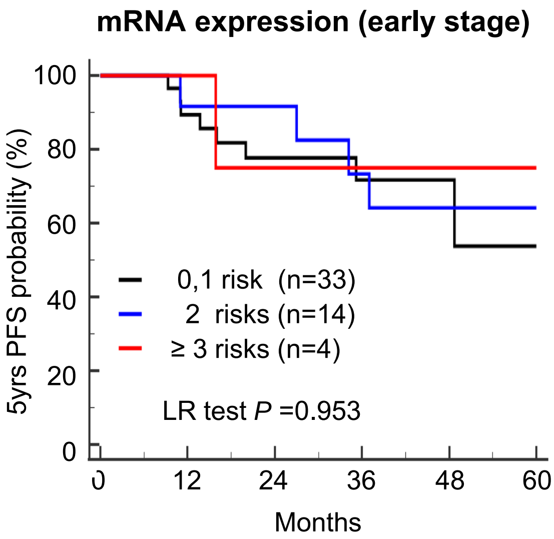

HR (95% CI) of 2 risks: 0.92 (0.28-3.05)

HR (95% CI) of ≥ 3 risks: 0.73 (0.11-4.72)

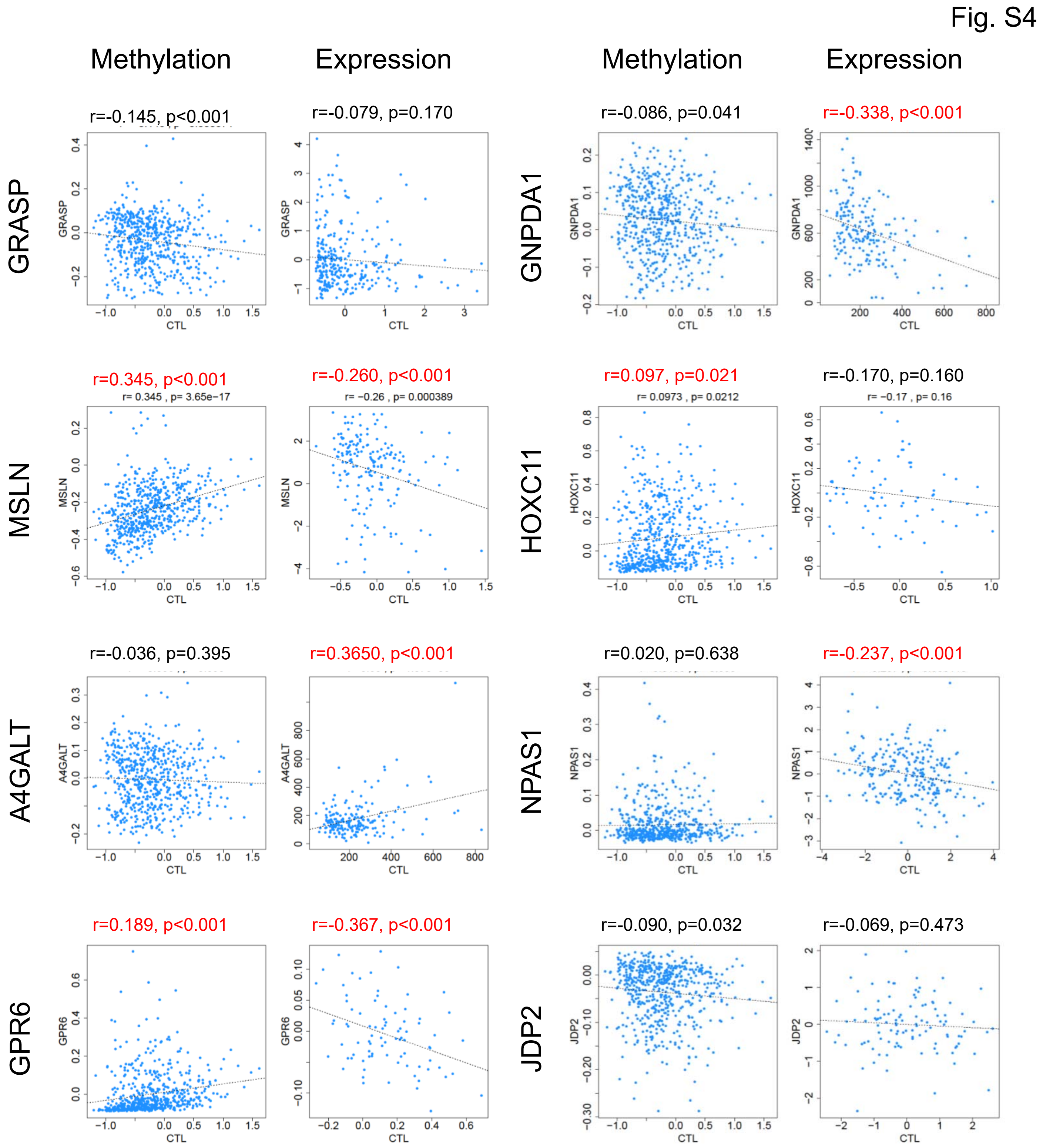

Fig. S4
Methylation
Expression
Methylation
Expression
GRASP
r=-0.145, p<0.001
r=-0.079, p=0.170
GNPDA1
r=-0.086, p=0.041
r=-0.338, p<0.001
MSLN
r=0.345, p<0.001
r= 0.345 , p= 3.65e−17
r=-0.260, p<0.001
r= −0.26 , p= 0.000389
HOXC11
r=0.097, p=0.021
r= 0.0973 , p= 0.0212
r=-0.170, p=0.160
r= −0.17 , p= 0.16
A4GALT
r=-0.036, p=0.395
r=0.3650, p<0.001
NPAS1
r=0.020, p=0.638
r=-0.237, p<0.001
GPR6
r=0.189, p<0.001
r=-0.367, p<0.001
JDP2
r=-0.090, p=0.032
r=-0.069, p=0.473
CTL

Fig. S5

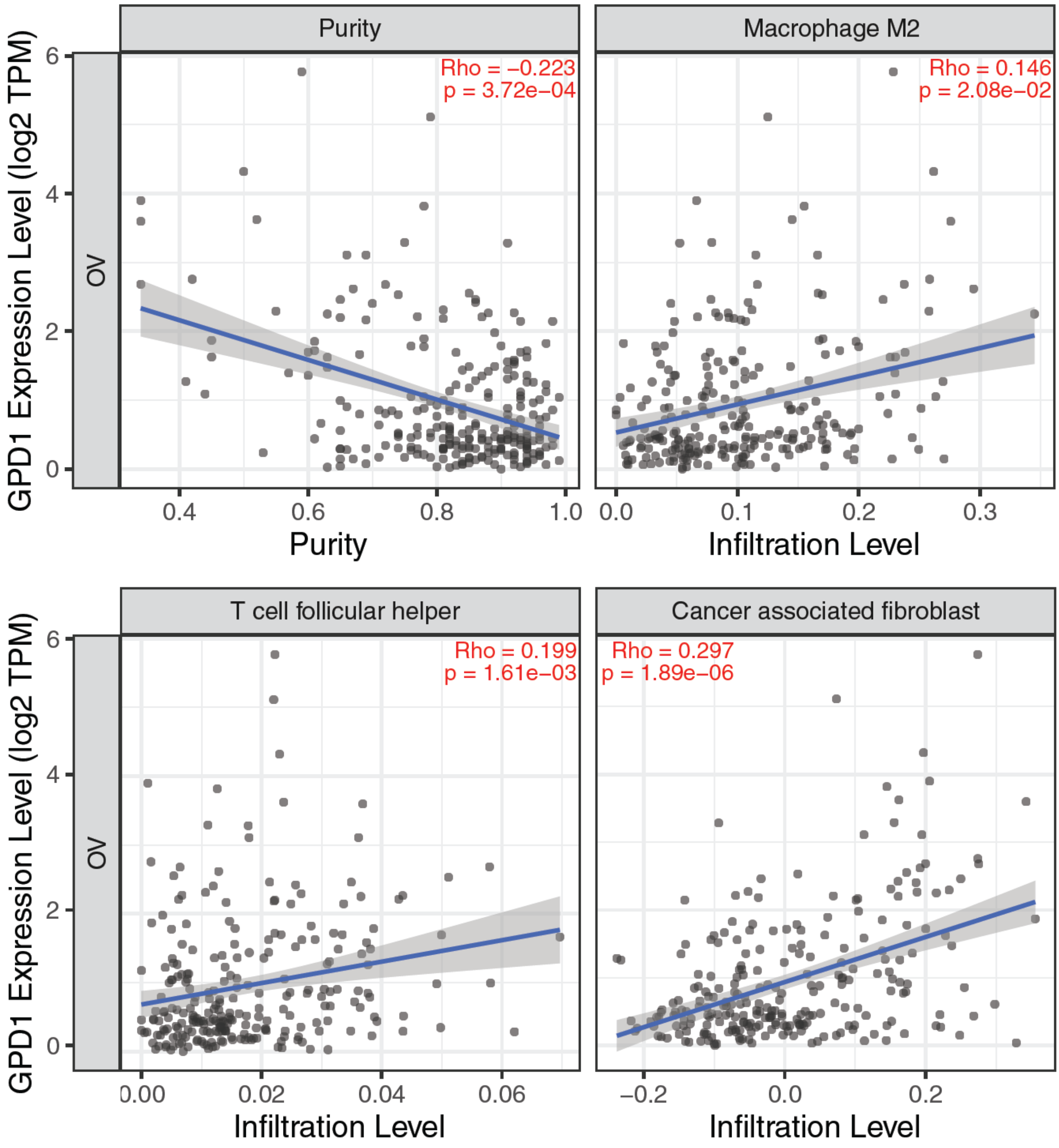

Purity
Macrophage M2
T cell follicular helper
Cancer associated fibroblast
OV
GPD1 Expression Level (log2 TPM)
Rho = −0.223
p = 3.72e−04
Rho = 0.146
p = 2.08e−02
Rho = 0.199
p = 1.61e−03
Rho = 0.297
p = 1.89e−06
Purity
Infiltration Level

Fig. S6

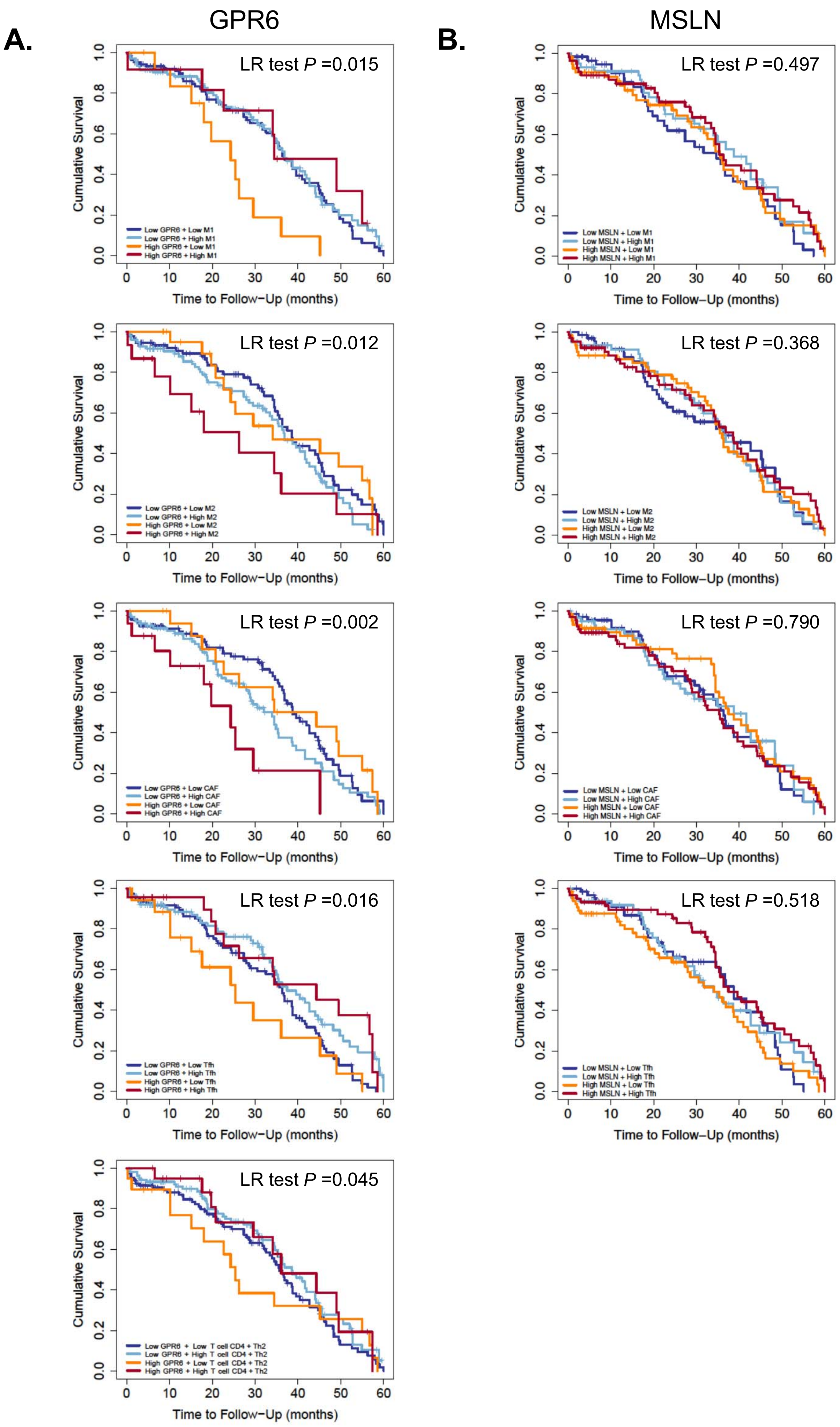
A.
GPR6
B.
MSLN
LR test P =0.015
Low GPR6 + Low M1
Low GPR6 + High M1
High GPR6 + Low M1
High GPR6 + High M1
LR test P =0.012
Low GPR6 + Low M2
Low GPR6 + High M2
High GPR6 + Low M2
High GPR6 + High M2
LR test P =0.002
Low GPR6 + Low CAF
Low GPR6 + High CAF
High GPR6 + Low CAF
High GPR6 + High CAF
LR test P =0.016
Low GPR6 + Low Tfh
Low GPR6 + High Tfh
High GPR6 + Low Tfh
High GPR6 + High Tfh
LR test P =0.045
Low GPR6 + Low T cell CD4 + Th2
Low GPR6 + High T cell CD4 + Th2
High GPR6 + Low T cell CD4 + Th2
High GPR6 + High T cell CD4 + Th2
LR test P =0.497
Low MSLN + Low M1
Low MSLN + High M1
High MSLN + Low M1
High MSLN + High M1
LR test P =0.368
Low MSLN + Low M2
Low MSLN + High M2
High MSLN + Low M2
High MSLN + High M2
LR test P =0.790
Low MSLN + Low CAF
Low MSLN + High CAF
High MSLN + Low CAF
High MSLN + High CAF
LR test P =0.518
Low MSLN + Low Tfh
Low MSLN + High Tfh
High MSLN + Low Tfh
High MSLN + High Tfh
Cumulative Survival
Time to Follow−Up (months)
