# Supplementary material for: Epigenomic Profiling of Epithelial Ovarian Cancer Stem-Cell Differentiation Reveals GPD1 Associated Immune Suppressive Microenvironment and Poor Prognosis
Source: Int J Mol Sci. 2022 May 4;23(9):5120. doi: 10.3390/ijms23095120 (PMC9101898; doi:10.3390/ijms23095120)
Supplement: Supplementary file 1 [file ijms-23-05120-s001.zip › Supplementary_20220403_IJMS.pdf]

Supplementary figure legends

Figure S1. A schematic diagram of epithelial ovarian cancer stem cells differentiation model.

(A) Epithelial ovarian cancer stem cell (SR1) is a ball shape with a smooth surface with high tumorigenicity, which has a translineage-differentiated capability. The progenitor stem cell (SR2) is irregular in shape with a morula-like surface with high tumorigenicity but showed no translineage-differentiated capability, only basic tumor cell morphology. The suspended SR1 and/or SR2 cells adhered and differentiated to attached cells in four different time points, early adhesion (AD1 and AD2) and late progression (AD3 and AD4). This OCSC differentiation model may represent cancer metastasis. (B) Phase A, cells from SR1 differentiated into SR2. Phase B, the suspended SR1 and/or SR2 cells adhered and differentiated to attach cells in two different time points. Phase C showed attached cell differentiation and proliferation in two different time points. (B) The flow chart of the analytical strategy. Methylome and transcriptome OCSCs were analyzed by MethylCap-seq and expression array. The differential methylated changes were selected by DMR ≥ 10%, and the differential expression genes were selected by │fold change│ ≥ 1.5 in three phases. The selected candidate genes were further assessed for survival relevance by recurrent risk score using the AOSC, TCGA, and KM-plotter databases. The genes associated with survival were annotated by protein–protein interaction and immune assessment.

Figure S2. The selection criteria of survival relevance of OCSC differentiation genes.

The recurrent risk score (RRS) of each gene was calculated by 15 criteria, which used five cutoff points multiplied by three different end points. When a gene's low methylation was associated with inferior survival, it would be counted as 1; when a gene's high methylation was associated with poor survival it would be scored as –1; if methylation was not associated with survival, it would be recorded as 0. The RRS has been summarized using the score of a total of 15 criteria. If both RRSs were above 1 or both less than –1 in the two databases, AOSC and TCGA, the gene would be selected. After this selection

process, we identified 11 genes with a methylation level that was significantly correlated with patients' survival.

Figure S3. The combination of the gene expression status of five-genes constituted an OCSC gene signature.

The combination of the gene expression status of 5 genes constituted an OCSC gene signature. Patients were grouped by 0–1 gene at risk (black line), 2 genes at risk (blue line), and ≥3 genes at risk (red lines) to determine the PFS. This gene signature was tested by expression of debulk status (A) and early-stage (B) of EOC patients by KM plotter. The *P*-value was calculated using the log-rank test.

Figure S4. The correlation between OCSC gene signature and cytotoxic T lymphocyte (CTL) level.

The correlation between gene methylation level and CTL level (left) as well as gene expression and CTL level (right) of each OCSC gene signature genes were estimated using TIDE algorithm.

Figure S5. The correlation between GPD1 expression level and different immune cell types infiltration level.

The correlation between GPD1 expression and tumor purity, M2 macrophage, T cell follicular helper cell, as well as cancer associated fibroblast in EOC using TIMER2.0 algorithm.

Figure S6. The association between immune infiltrates and clinical outcome of GPR6 and MSLN expression level of EOC patients.

The 5-year overall survival of GPR6 (A) and MSLN (B) mRNA expression levels with M1/M2 macrophage, Tfh, CAF, and T cell CD4 + Th2 cells in EOC patients was assessed using the TCGA database.
